# Health‐Related Performance Decrements Resulted in Substantial Financial Loss for Team USA Athletes at the Paris 2024 Olympic Games

**DOI:** 10.1002/ars2.70132

**Published:** 2026-10-27

**Authors:** Jacob F. Oeding, Travis Anderson, Ashley N. Triplett, Amber Donaldson, Jonathan T. Finnoff, Heather K. Vincent, Eric G. Post

**Affiliations:** ^1^ Department of Physical Medicine and Rehabilitation University of Utah Salt Lake City Utah U.S.A.; ^2^ Department of Sports Medicine United States Olympic & Paralympic Committee Colorado Springs Colorado U.S.A.; ^3^ United States Coalition for the Prevention of Illness and Injury in Sport Colorado Springs Colorado U.S.A.; ^4^ U.S. Soccer Fayetteville Georgia U.S.A.; ^5^ University of Colorado School of Medicine Denver Colorado U.S.A.

## Abstract

**Purpose:**

To estimate the financial impact of injury and illness among Team USA athletes who participated in the Paris 2024 Olympic Games and evaluate the cost‐effectiveness of a feasible illness prevention program.

**Methods:**

All Team USA athlete‐events at the Paris 2024 Olympic Games were analyzed. Precompetition betting markets were used to derive the expected performance. Counterfactual finish distributions were generated using a Markov ranking model. Injury‐ and illness‐associated performance decrements were estimated relative to expected medal placement outcomes and translated into expected financial losses across 3 domains: Operation Gold performance incentives, athlete stipends, and estimated commercial value. Losses were summarized at the athlete‐event, athlete, and Team USA levels. A cohort Markov cost‐effectiveness model evaluated a potential illness prevention program using probabilistic sensitivity analysis.

**Results:**

Across 808 Team USA athlete‐events, 186 injuries and 108 illnesses were recorded. Total injury‐ and illness‐attributable financial losses were estimated at $1,014,210 under conservative commercial value assumptions (*B* = $10,000), including $462,409 attributable to injuries and $551,802 attributable to illnesses. Median athlete‐event‐level financial loss was $3000 (75th percentile: $5094; 90th percentile: $7758), whereas median athlete‐level loss was $4710 (90th percentile: $15,617; maximum: $62,915), indicating substantial right skewness and concentration of losses. Illness events were associated with higher mean financial losses per event than injuries ($5109 vs $2486). An illness prevention program costing $200 per athlete and reducing illness risk by 40% was cost‐saving, averting $220,721 in expected losses at a total cost of $117,000, yielding net savings of $103,721, and remaining cost‐saving in 95.2% of probabilistic simulations.

**Conclusions:**

Injury and illness at the Olympic Games are associated with substantial and highly concentrated financial losses, with illnesses accounting for a disproportionate share of the total impact on Team USA athletes.

**Level of Evidence:**

Level IV, retrospective cohort study with counterfactual performance modeling.

## Graphical abstract

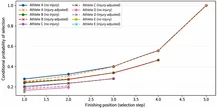

Injury and illness are persistent challenges for athletes competing at the Olympic Games.^1^, ^2^, ^3^, ^4^ Large, prospective surveillance programs conducted during multiple Olympic cycles have shown that 8% to 15% of athletes sustain at least 1 injury and 4% to 12% experience illness during the Games period, with substantial variation across sports, events, and athlete subgroups.^2^, ^3^, ^4^ These health events can reduce training and competition availability, impair readiness, and, in some cases, contribute to withdrawal or noncompletion, underscoring their potential to influence competitive outcomes in high‐stakes environments.^1^, ^2^, ^3^, ^4^

In parallel with ongoing surveillance work, research has increasingly examined the consequences of injury and illness beyond incidence alone.^1^, ^5^, ^6^, ^7^, ^8^, ^9^, ^10^, ^11^ Within Team USA specifically, a series of studies has characterized injury/illness patterns across major multisport events (e.g., Olympic and Paralympic Games, Pan American Games, and Youth Olympic Games), including the development and implementation of standardized surveillance infrastructure to support systematic capture of time‐loss and nontime‐loss health problems.^5^, ^6^, ^7^, ^8^, ^9^, ^10^, ^11^ Extending this line of work, Anderson et al. showed that injury and illness among Team USA at the Paris 2024 Olympic Games were associated with underperformance relative to ex ante finishing position expectations and with altered probabilities of achieving key competitive outcomes such as medaling.^10^ More broadly, meta‐analytic evidence synthesizing Olympic, Youth Olympic, and Paralympic datasets reinforces that injury and illness epidemiology is context‐specific and that respiratory illness is a recurrent contributor across Games settings, highlighting persistent prevention targets.^12^

Olympic performance is closely tied to financial outcomes. For many athletes, finishing position influences eligibility for direct incentive programs, ongoing support mechanisms, and broader commercial opportunities linked to visibility and public attention during major events.^13^, ^14^, ^15^, ^16^ For example, Operation Gold is a financial incentive program by the U.S. Olympic & Paralympic Committee (USOPC) that provides monetary rewards to elite athletes for the top finishes at the Olympic and Paralympic Games. Further monetary stipends are also provided to Team USA athletes by sport national governing bodies or the USOPC. Commercial value is increasingly shaped by athletes’ ability to leverage digital platforms and personal branding, with evidence indicating that athletic success and on‐field credibility remain central drivers of endorsement and brand equity in many contexts.^13^, ^14^ Importantly, these financial consequences are often threshold‐based (e.g., medal vs nonmedal and top‐N cut points). As such, small health‐related performance decrements near qualification cutoffs may translate into disproportionate changes in financial returns.^15^, ^17^ In other words, financial risk may be unevenly distributed, particularly among medal‐contending athletes and athletes in sports with greater market visibility, for whom small health‐related changes in finishing position may disproportionately affect Operation Gold payments, stipend eligibility, and commercial opportunities.

Despite these links between health, performance, and financial outcomes, the economic implications of injury and illness at the Olympic Games are not well understood, and little is known about how health‐related performance decrements translate into the financial risk for athletes and associated organizations (e.g., USOPC and national governing bodies).^10^ Moreover, financial risk is likely to be unevenly distributed because athletes with higher expected performance and greater visibility may have substantially larger exposure to threshold‐driven incentives and commercial value dynamics.^13^, ^14^, ^15^

The purposes of this study were to estimate the financial impact of injury and illness among Team USA athletes who participated in the Paris 2024 Olympic Games and evaluate the cost‐effectiveness of a feasible illness prevention program. We hypothesized that injury and illness would be associated with substantial financial losses concentrated among a small subset of athletes and that a targeted illness prevention program would be cost‐saving under plausible assumptions.

## METHODS

### Data and Cohort Definition

The study population consisted of all Team USA athletes who represented the United States at the Paris 2024 Summer Olympic Games. To generate accurate ex ante performance expectations, including the derivation of percentile‐based finishing positions, performance data from the full field of competitors across all Paris 2024 Olympic events were incorporated into the modeling framework. Because detailed injury and illness information was available exclusively for Team USA, inferential analyses were restricted to these Olympic participants.

### Ethical Considerations

Ethical oversight for this investigation was provided by the Institutional Review Board at the University of Utah (IRB no. 00187889). All study procedures were conducted in compliance with applicable ethical standards and regulatory requirements. Because of the sensitive nature of the Team USA athlete health and performance information, the datasets analyzed in this study are not publicly accessible.

### Injury and Illness Surveillance

During the Paris 2024 Summer Olympic Games, health events among Team USA athletes were documented using the USOPC's Injury and Illness Surveillance reporting methodology.^5^ Medical personnel embedded with Team USA logged all reportable injuries and illnesses occurring during the full surveillance window, which encompassed an 11‐day precompetition phase, the official competition period, and a post‐Games phase (July 15‐26, 2024; July 27‐August 11, 2024; and August 12‐15, 2024, respectively). The surveillance protocol, described in detail in prior methodological work,^5^ required clinicians to record every medically diagnosed injury or illness, regardless of whether it resulted in time loss from training or competition. Each entry included the date of symptom onset, a standardized diagnostic classification, and additional clinical descriptors relevant to the condition. To promote data completeness and accuracy, records submitted by venue and team medical staff were reviewed daily by the USOPC Sports Medicine Research team and systematically cross‐checked against the electronic medical record system.

### Market‐Based Expectation Model

Sports betting odds data recorded from a publicly facing website of a commercial betting company were used to derive ex ante performance expectations for Team USA athletes. Complete methodology and rationale have been previously described by Anderson et al.^10^ For each athlete‐event, implied win probabilities were used as ex ante performance forecasts. Missing win probabilities were imputed using rank‐wise mean imputation (the mean probability among all competitors with the same finishing rank).

### Performance Modeling

Win probabilities were calibrated to observed rankings, and event‐specific finish distributions were generated using 5000 Plackett‐Luce simulations. Injury/illness‐related performance decrements were estimated from expected and observed percentile finish and applied as nonpositive rank penalties to generate counterfactual no‐health‐event and health‐adjusted distributions. Full model specifications are provided in the Supporting Information: Appendix I.

### Financial Impact Models: Overview and Counterfactual Framework

The financial consequences of injury and illness were quantified using a counterfactual expected‐value framework. For each athlete‐event entry, 2 parallel outcome distributions were constructed: (1) a counterfactual no‐injury distribution representing expected financial outcomes had the athlete competed without injury or illness and (2) an injury‐adjusted distribution incorporating the estimated performance decrement associated with injury or illness. Financial loss attributable to injury or illness was defined as the difference in the expected financial value between these 2 distributions. All analyses were conducted at the athlete‐event level and subsequently aggregated to the athlete and Team USA levels.

### Financial Impact Models: Expected Medal Finish Distributions

Event‐specific finish distributions were generated using a calibrated Markov ranking model equivalent to the Plackett‐Luce process. For each event, simulated rankings yielded probabilities of medal finishes (gold, silver, and bronze) and threshold finishes (top 5, top 8, and top 10). These probabilities formed the basis for all expected‐value calculations. Injury‐adjusted distributions were produced by applying an empirically estimated injury‐related rank penalty to each simulated draw, preserving event‐level uncertainty while shifting expected performance downward in injured or ill athletes.

Figure 1 provides an illustrative example of the Markov ranking model, comparing no‐injury versus injury‐adjusted stepwise selection probabilities for an individual athlete‐event. At each finishing position (selection step), probabilities represent the conditional likelihood that a given athlete is assigned that position, given which athletes remain unassigned. Injury reduces the probability that the affected athlete is selected at early finishing positions, with probability mass redistributed among remaining competitors. These shifts propagate sequentially across subsequent finishing positions, illustrating how small injury‐related performance penalties can generate nonlinear changes in medal and near‐medal probabilities within the Markov ranking framework.

**FIGURE 1:**
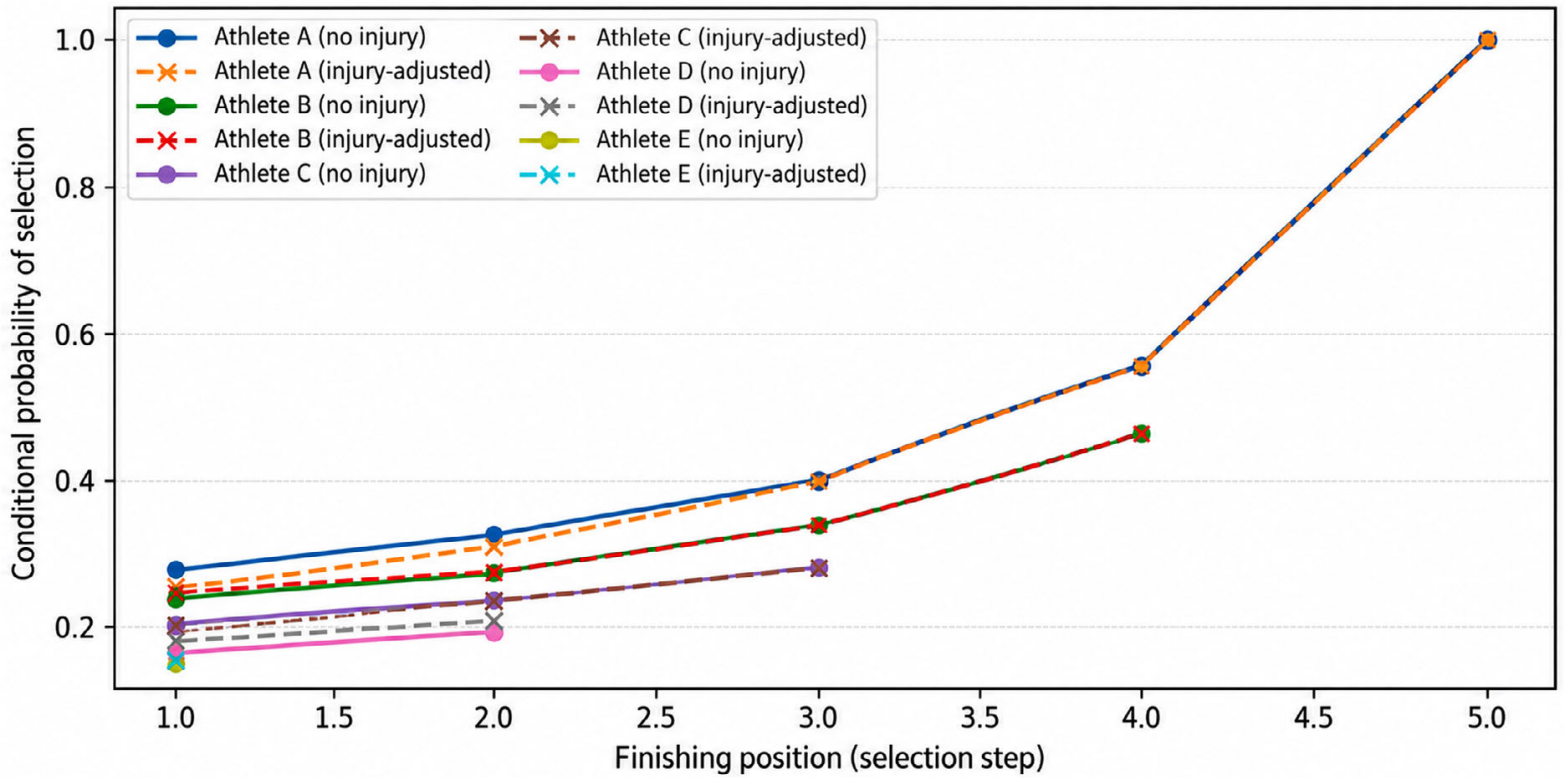
Markov ranking model: comparison of no‐injury versus injury‐adjusted stepwise selection probabilities for an individual event (e.g., men's 100‐m dash). Solid lines show stepwise selection probabilities under a no‐injury scenario, and dashed lines show probabilities after applying an injury‐related performance decrement to the highest‐ranked athlete. At each finishing position (selection step), probabilities represent the conditional likelihood that a given athlete is assigned that position, given which athletes remain unassigned. Injury reduces the probability that the affected athlete is selected at early finishing positions, with probability mass redistributed among remaining competitors. These shifts propagate sequentially across subsequent finishing positions, illustrating how small injury‐related performance penalties can generate nonlinear changes in medal and near‐medal probabilities within the Markov ranking framework.

### Financial Impact Models: Operation Gold Valuation

The expected Operation Gold value for each athlete‐event was computed using actual current financial stipends provided for each medal placement:

$${\text{EV}}_{\text{OG}}=37,500\cdot \text{Pr}\left(\text{gold}\right)+22,500\cdot \text{Pr}\left(\text{silver}\right)+15,000\cdot \text{Pr}\left(\text{bronze}\right)$$

The observed Operation Gold value was determined from documented finishing placement at the games. Injury‐attributable Operation Gold loss was defined as the difference between the expected no‐injury value and injury‐adjusted expected value. Athletes finishing outside the top 3 were assigned a value of 0.

### Financial Impact Models: Athlete Stipend Valuation

Athlete stipends were modeled using sport‐specific qualification thresholds (medal, top 5, top 8, or top 10). For each athlete‐event, the probability of meeting the applicable threshold was computed from simulated finish distributions. The expected stipend value was calculated as the stipend amount multiplied by the probability of qualification. Injury‐attributable stipend loss was defined analogously to Operation Gold as the difference between no‐injury and injury‐adjusted expected values.

### Financial Impact Models: Commercial Value Estimation

Commercial value was modeled as a probabilistic function of performance and sport‐level visibility. A Market Visibility Index (MVI) was constructed for each sport by normalizing the volume of available betting lines and competitive entries, scaled from 0 to 1. For each athlete‐event, expected commercial value was computed as

$${\text{EV}}_{\text{Comm}}=B\cdot \left(0.5+{\text{MVI}}_{\text{Sport}}\right)\cdot E\left[\text{Performance}\;\text{Multiplier}\right]$$

where *B* represents a base commercial value parameter and performance multipliers were prespecified as heuristic weights representing a monotonic, nonlinear increase in commercial value with higher Olympic placement (6.0 for gold medal finishes, 4.0 for silver, 3.0 for bronze, 1.5 for nonmedal top‐8 finishes, 1.2 for 9th‐10th place finishes, and 1.0 otherwise). Sensitivity analyses were conducted across *B* = $3000, $10,000, and $30,000. Injury‐attributable commercial loss was defined as the difference between no‐injury and injury‐adjusted expected commercial value.

### Financial Impact Models: Aggregation and Uncertainty

Financial losses were aggregated across athlete‐events to generate athlete‐level and Team USA‐level estimates. Uncertainty was propagated through Monte Carlo simulation, with 5000 iterations per event. The reported results represent mean expected losses; distributions were retained for secondary analyses examining variance and tail risk.

### Injury‐ and Illness‐Specific Financial Losses

Injury and illness were modeled separately to estimate their respective performance decrements. Event‐specific penalties were applied to simulated finish distributions, and Operation Gold, stipend, and commercial losses were recalculated for each health event type. Attribution was assessed across commercial base values of $3000, $10,000, and $30,000. Full equations and procedures are provided in the Supporting Information: Appendix II.

### Cost‐Effectiveness Analysis of a Prospective Illness Prevention Program

A cost‐effectiveness analysis was conducted to evaluate whether a feasible health prevention program could reduce injury/illness‐attributable financial losses at the Paris 2024 Olympic Games. A cohort Markov model was constructed with cycle length defined as an athlete‐event exposure, consistent with the athlete‐event unit used in the primary performance and financial analyses. The analytical perspective was the organizational financial loss perspective, quantifying prevention value as reductions in expected injury/illness‐attributable losses (Operation Gold, stipends, and commercial value). The time horizon was 1 Games cycle (Paris 2024), represented as the observed distribution of athlete‐event exposures.

The cohort Markov model assigned athlete‐event exposures to no health event, injury, or illness states using observed frequencies and state‐specific financial losses. An illustrative respiratory illness prevention program was modeled as reducing illness risk without affecting injury risk and incurring a fixed per‐athlete cost. Outcomes included losses averted, net savings, benefit‐cost ratio, and cost per dollar of loss averted, with heterogeneity assessed across expected‐performance quartiles. Uncertainty was evaluated using 20,000 probabilistic sensitivity analysis iterations. Full model assumptions, equations, and parameter distributions are provided in the Supporting Information: Appendix III.

## RESULTS

### Sample

The Team USA cohort included 808 athlete‐event entries. Injury/illness was documented for 294 athlete‐event entries (36.4%).

### Performance Impact of Injury/Illness

The percentile performance model showed a statistically significant negative shift associated with injury/illness, with a significant interaction (*P* < .001) indicating that the magnitude of the decrement varies with the expected percentile performance. The fitted coefficients are provided in Table 1.

**TABLE 1:** Fitted Coefficients of Percentile Performance Model

Term	Estimate	SE_HC3_	*P* Value
Intercept	9.51	3.41	.005
Expected percentile	0.92	0.05	<.001
Injury/illness	−16.61	5.50	.003
Expected √ó injury/illness	0.22	0.08	.009

Figure 2 depicts the relation between the expected and observed percentile finish by injury/illness status. It is important to note that the fitted interaction between expected percentile finish and injury/illness indicates that injury‐related performance decrements are largest among athletes with lower ex ante expectations and attenuate among athletes with very high expected performance. In some cases, injured or ill athletes with very high expected percentiles exhibited observed placements comparable with or slightly better than their noninjured counterparts. This pattern likely reflects selection and ceiling effects: only athletes with relatively mild health impairments are able to compete at the highest expected performance levels, and observed percentile outcomes are bounded above. To account for this, the statistical model allowed the effect of injury/illness to vary across the expected performance distribution, and counterfactual simulations constrained injury effects to be nonpositive so that injury or illness could not artificially improve expected outcomes.

**FIGURE 2:**
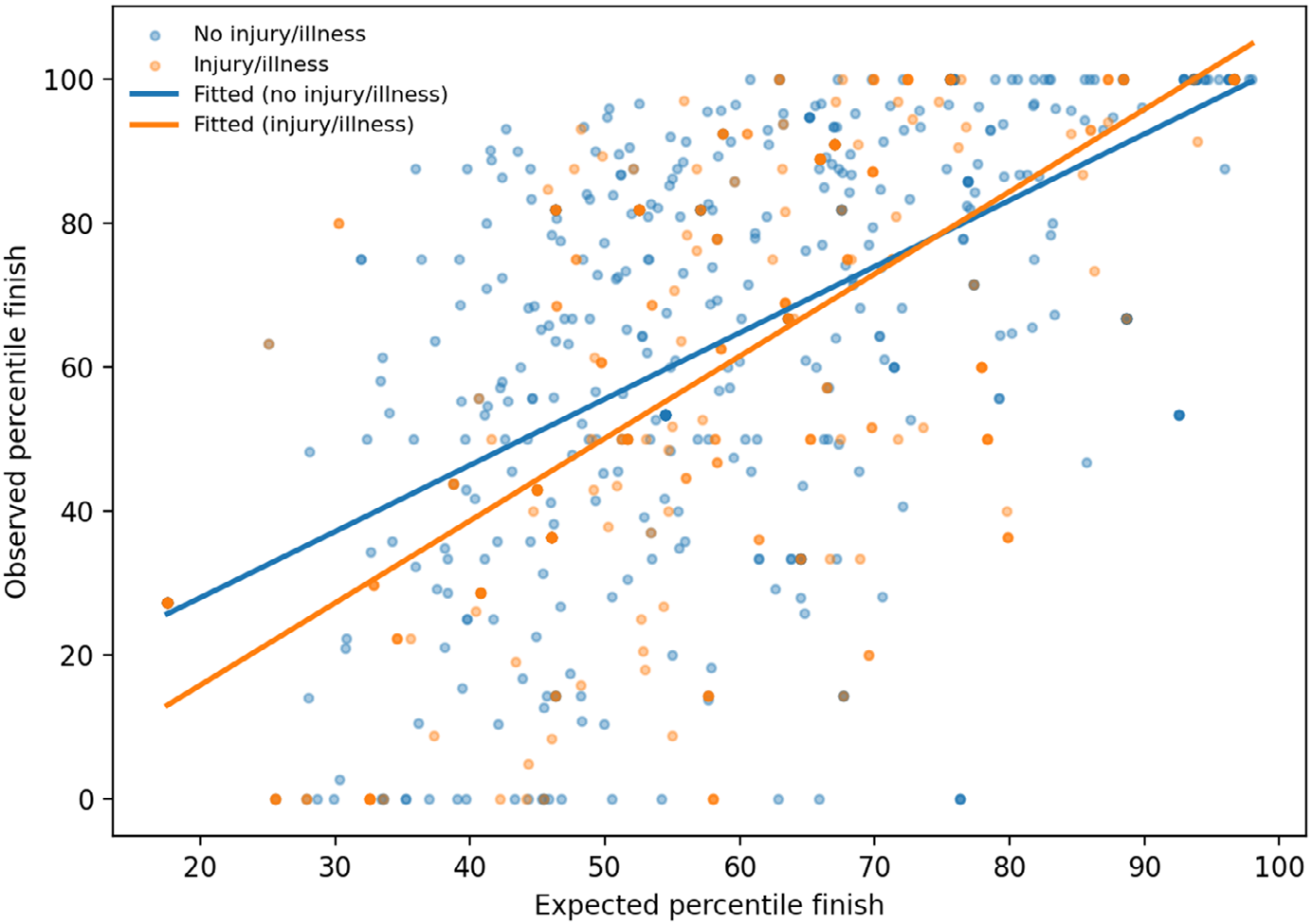
Relation between expected and observed percentile finish by injury/illness status—scatterplot showing observed percentile finish as a function of expected (ex ante) percentile finish for Team USA athlete‐event entries at the Paris 2024 Olympic Games. Each point represents a single athlete‐event observation, with blue points indicating athletes without a documented injury or illness and orange points indicating athletes with a documented injury or illness. Solid lines depict fitted values from the interaction model relating observed percentile finish to expected percentile finish, stratified by injury/illness status.

### Financial Losses Attributable to Injury/Illness

When aggregated across all injured athlete‐event entries, the estimated total injury‐ and illness‐attributable financial loss for Team USA was $1,051,034 under the primary commercial‐value specification (*B* = $10,000). Financial losses were distributed across 3 primary categories: Operation Gold performance incentives, athlete stipends, and estimated commercial value.

#### Operation Gold Losses

Injury‐ and illness‐related performance decrements resulted in an estimated $327,679 loss in expected Operation Gold payments. These losses arose primarily from reductions in medal probabilities among athletes with high ex ante medal likelihoods. Because Operation Gold awards are discrete and tiered, relatively small injury‐related shifts in expected placement near medal cutoffs translated into substantial financial losses. Losses were largest among athletes expected to finish in medal positions prior to injury or illness.

#### Athlete Stipend Losses

Estimated losses attributable to missed or reduced stipend eligibility totaled $82,189. Stipend losses were driven largely by athletes whose injury‐adjusted performance distributions shifted below sport‐specific qualification thresholds (e.g., medal, top‐5, top‐8, or top‐10 cutoffs). Although individual stipend amounts were smaller than Operation Gold awards, stipend losses were distributed across a larger number of athlete‐event entries, contributing meaningfully to the aggregate financial impact.

#### Commercial Value Losses

Estimated losses in commercial value totaled $641,166 under the primary specification (*B* = $10,000). Commercial losses reflected reductions in expected medal and high‐placement finishes, weighted by sport‐level visibility. Although commercial value estimates are inherently uncertain, these losses represented the largest single component of the total financial impact, accounting for approximately 61% of aggregate losses. Commercial losses scaled approximately linearly with the magnitude of injury‐related performance decrements.

### Distribution and Concentration of Financial Losses

Financial losses were highly concentrated across athletes. When aggregated to the athlete level, the distribution of total injury‐attributable losses was extremely right‐skewed. The Gini coefficient for athlete‐level total financial loss was 0.88, indicating pronounced inequality in financial impact. The top 20% of athletes accounted for approximately 99% of total injury‐related financial losses, reflecting the concentration of financial incentives among a relatively small subset of medal‐contending athletes. This concentration was driven primarily by Operation Gold and commercial‐value losses among athletes with high expected performance entering the Games.

### Scenario Analyses

Scenario analyses were conducted to assess the sensitivity of financial losses to the magnitude of injury‐related performance decrements. Under a low‐impact scenario in which injury‐related performance penalties were reduced by 50%, total Team USA financial losses were estimated at $530,446, including $165,398 in Operation Gold losses, $37,558 in stipend losses, and $327,490 in commercial losses. Under the baseline scenario, reflecting empirically estimated injury effects, total losses were $1,051,034, with $327,679 attributable to Operation Gold, $82,189 to stipends, and $641,166 to commercial value. Under a high‐impact scenario assuming a 150% scaling of injury‐related performance penalties, total losses increased to $1,453,759, including $447,960 in Operation Gold losses, $120,816 in stipend losses, and $884,983 in commercial losses. Increases in total losses under the high‐impact scenario were driven disproportionately by Operation Gold and stipend losses, reflecting nonlinear threshold effects associated with medal and placement cutoffs.

### Sensitivity to Commercial Value Assumptions

Sensitivity analyses varying the base commercial value parameter showed proportional effects on estimated commercial losses while leaving Operation Gold and stipend losses unchanged (Table 2). Under a lower commercial‐value specification (*B* = $3,000), total injury‐attributable losses were substantially smaller, whereas, under a higher specification (*B* = $30,000), commercial losses dominated the aggregate financial impact. Across all specifications, however, the relative concentration of losses among a small subset of athletes and the prominence of threshold‐based financial effects remained consistent.

**TABLE 2:** Estimated Injury‐ and Illness‐Attributable Financial Losses for Team USA at the Paris 2024 Olympic Games∗

Financial Component	Commercial Base = $3000	Commercial Base = $10,000	Commercial Base = $30,000
Operation Gold losses (USD)	327,679	327,679	327,679
Athlete stipend losses (USD)	82,189	82,189	82,189
Commercial value losses (USD)	192,350	641,166	1,923,498
Total financial loss (USD)	602,218	1,051,034	2,333,366
Commercial losses as % of total	31.9%	61.0%	82.4%

∗ Financial losses represent the difference between counterfactual no‐injury expected values and injury‐adjusted expected values, summed across all injured athlete‐event entries. Operation Gold and stipend losses are invariant across commercial‐value specifications. Commercial losses scale linearly with the base commercial value parameter. All estimates are derived from injury‐adjusted simulated finish distributions using an event‐level Markov ranking model.

### Percentage of Financial Losses Attributable to Injuries Versus Illnesses

Across 808 Team USA athlete‐event entries, 186 entries were classified as injuries and 108 as illnesses (the remainder had no documented health event) (Figure 3). Figure 4 displays the estimated marginal effect of injury (blue) and illness (orange) on observed percentile finish as a function of expected percentile finish, derived from the interaction model. The shaded regions represent 95% confidence intervals. Negative values indicate performance decrements relative to no health event. Injury effects are relatively consistent across the performance distribution, whereas illness effects are larger at lower expected percentiles and attenuate toward 0 among higher expected performers.

**FIGURE 3:**
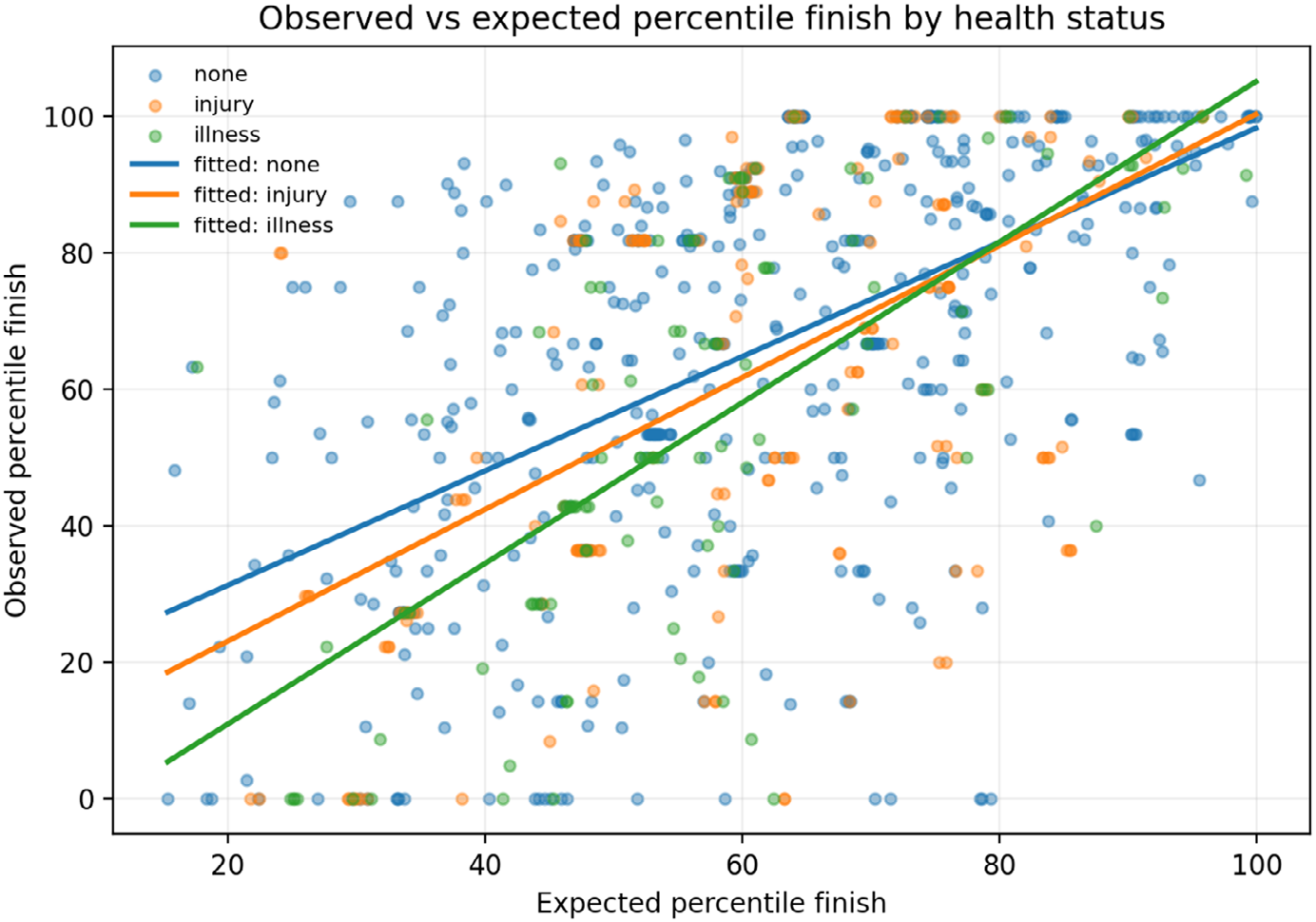
Observed versus expected percentile finish by health status scatter plot showing observed percentile finish plotted against expected (ex ante) percentile finish for Team USA athlete‐event entries, stratified by health status (no injury/illness, injury, and illness). Solid lines represent fitted linear relations from the regression model including expected percentile, health status indicators, and interaction terms. Deviations below the identity line indicate underperformance relative to expectation. Injury and illness are associated with downward shifts in observed performance relative to no health event, with effect magnitudes varying across the expected performance distribution.

**FIGURE 4:**
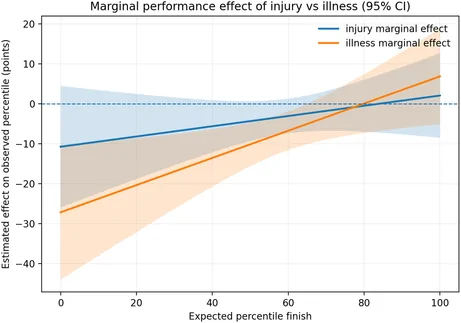
Marginal performance effect of injury versus illness across expected performance. (CI, confidence interval.)

Using the injury‐ and illness‐specific performance penalty model applied within the Markov ranking simulations, the total injury/illness‐attributable financial loss under the primary commercial value specification (*B* = $10,000) was $1,014,480 (Figure 5). Of this total, injuries accounted for $458,485 (45.19% of total financial loss), and illnesses accounted for $555,994 (54.81% of total financial loss). Component‐level attribution (*B* = $10,000) is provided in Table 3.

**FIGURE 5:**
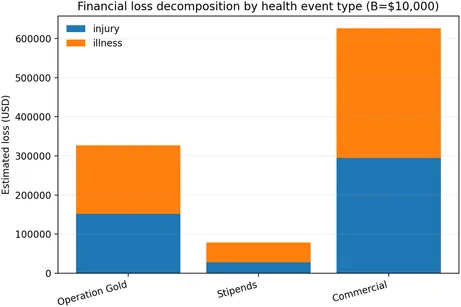
Financial loss decomposition by health event type (*B* = $10,000). Stacked bar chart showing estimated injury‐ and illness‐attributable financial losses by component: Operation Gold, athlete stipends, and commercial value. Losses are computed as the difference between no‐health‐event and health‐adjusted expected values derived from simulated finish distributions. Commercial losses represent the largest component for both injuries and illnesses under the primary commercial value specification.

**TABLE 3:** Component‐Level Attribution of Injury‐ and Illness‐Attributable Financial Losses for Team USA (B = $10,000)∗

Financial Component	Total Loss (USD)	Injury‐Attributable Loss (USD)	Injury‐Attributable, %	Illness‐Attributable Loss (USD)	Illness‐Attributable, %
Operation Gold	321,098	146,967	45.77%	174,130	54.23%
Athlete stipends	77,916	26,643	34.19%	51,273	65.81%
Commercial value	615,466	284,875	46.29%	330,591	53.71%
Total	1,014,480	458,485	45.19%	555,994	54.81%

∗ Commercial value losses are estimated using a base commercial value parameter of *B* = $10,000. Percentages represent the share of total component‐level losses attributable to injuries or illnesses, respectively.

The robustness across commercial value assumptions, with the percent of total loss attributable to injuries versus illnesses, is provided in Table 4. Attribution percentages were stable across commercial base value parameters.

**TABLE 4:** Robustness of Injury Versus Illness Attribution Across Commercial Value Assumptions∗

Commercial Base Value (*B*)	Total Financial Loss (USD)	Injury‐Attributable Loss, %	Illness‐Attributable Loss, %
$3000	583,653	44.39%	55.61%
$10,000	1,014,480	45.19%	54.81%
$30,000	2,245,412	45.79%	54.21%

∗ Total financial loss represents the sum of injury‐ and illness‐attributable losses across Operation Gold, athlete stipends, and commercial value. Percentages indicate the share of total loss attributable to injuries or illnesses under each commercial base value specification.

### Athlete‐Event Financial Impact

Figure 6 illustrates the distribution of injury‐ and illness‐attributable financial losses at the athlete‐event level. Financial losses were highly right‐skewed for both health event types, with a small number of athlete‐event entries accounting for disproportionately large losses. Although illnesses occurred less frequently than injuries, illness‐associated losses exhibited a higher median and greater upper‐tail dispersion, indicating larger average financial losses per affected event. This pattern reflects the tendency for illnesses to disproportionately affect athletes with higher expected performance and greater financial exposure, amplifying threshold‐based losses despite a lower overall incidence.

**FIGURE 6:**
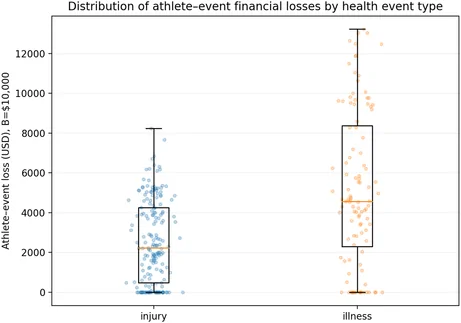
Distribution of athlete‐event financial losses by health event type. Distribution of estimated athlete‐event‐level financial losses for injuries and illnesses (commercial base value *B* = $10,000). Points represent individual athlete‐event entries; boxplots summarize the median, interquartile range, and overall dispersion. Both distributions are right‐skewed, with illnesses exhibiting a higher median and greater upper‐tail dispersion, reflecting larger average financial losses per event despite fewer total illness events.

Across all health‐affected athlete‐event entries (N = 294), the median athlete‐event loss was $3000, with a 75th percentile of $5094 and a 90th percentile of $7758 (95th percentile: $9764; maximum: $12,713) (Table 5). These results indicate that for many injured or ill athletes, financial consequences were relatively modest, particularly when athletes were not positioned near medal or stipend thresholds. However, the upper tail of the distribution was pronounced. Illness‐associated athlete‐event losses exhibited greater dispersion than injury‐associated losses, consistent with the finding that illnesses disproportionately affected athletes with higher expected performance and greater financial exposure. This pattern reflects the amplification of relatively small performance decrements when athletes are near discrete incentive thresholds.

**TABLE 5:** Summary of Athlete‐Event and Athlete‐Level Financial Losses Attributable to Injury and Illness at the Paris 2024 Olympic Games∗

Analysis Level/Subgroup	N	25th Percentile (USD)	Median (USD)	75th Percentile (USD)	90th Percentile (USD)	95th Percentile (USD)	Maximum (USD)
Athlete‐event level (all health events)	294	1642	3000	5094	7758	9764	12,713
Athlete level (aggregated across events)	153	2981	4710	9266	15,617	20,941	62,915
Expected‐performance quartiles (athlete‐event level)							
Q1 (lowest expected percentile)	74	2114	3612	5946	8417	9882	12,190
Q2	73	2378	4095	6821	9906	11,742	12,713
Q3	73	1921	3244	5103	7631	9388	11,806
Q4 (highest expected percentile)	74	0	0	2844	4761	6118	7685

∗ All values represent expected financial losses attributable to injury or illness, calculated as the difference between no‐health‐event and health‐adjusted expected values derived from event‐level Markov ranking simulations. Athlete‐event losses reflect single competition entries, whereas athlete‐level losses aggregate losses across all affected events for each athlete. Expected‐performance quartiles are defined by quartiles of expected percentile finish among health‐affected athlete‐event entries.

### Athlete‐Level Financial Impact

When losses were aggregated across individual athletes (summing across all affected events for each athlete), the skewness of the distribution became more pronounced. Among athletes with at least 1 health‐affected entry (N = 153), the median athlete‐level loss was $4710, with a 75th percentile of $9266 and a 90th percentile of $15,617 (95th percentile: $20,941; maximum athlete‐level loss: $62,915). These results indicate that although most athletes experienced limited direct financial impact, a smaller subset of athletes incurred substantially larger expected losses because of the cumulative effects of health events across one or more competitions.

### Losses Conditional on Expected‐Performance Tier

Financial losses varied systematically across expected‐performance tiers. When athlete‐event entries were stratified into quartiles based on expected percentile finish, losses were largest in the middle tiers and smaller in the highest expected‐performance quartile. In the top expected‐performance quartile, the median athlete‐event loss was $0, with a 90th percentile of $4761 (maximum: $7685). This pattern reflects attenuation of the estimated injury and illness effects at higher expected percentiles in the fitted interaction model, as well as the application of a 1‐sided constraint preventing health events from improving the simulated performance. These conditional analyses highlight that financial consequences are not determined solely by the presence of a health event but by the interaction between health status and competitive context, particularly proximity to medal and incentive thresholds.

### Sensitivity to Commercial Value Assumptions

Because commercial value constitutes the most variable component of the total financial impact and scales linearly with the base commercial value parameter, athlete‐level losses increased substantially under higher commercial value specifications (Table 6). Under *B* = $30,000, the median athlete‐level loss increased to approximately $9995, the 90th percentile increased to approximately $35,240, and the maximum athlete‐level loss exceeded $140,000. These findings indicate that under plausible higher commercial value assumptions, injury and illness can be associated with expected financial losses on the order of tens to hundreds of thousands of dollars for a small subset of athletes with high expected performance and visibility.

**TABLE 6:** Sensitivity Analysis: Distribution of Athlete‐Event and Athlete‐Level Financial Losses Attributable to Injury and Illness (*B* = $30,000)∗

Analysis Level/Subgroup	N	25th Percentile (USD)	Median (USD)	75th Percentile (USD)	90th Percentile (USD)	95th Percentile (USD)	Maximum (USD)
Athlete‐event level (all health events)	294	3981	7812	14,248	21,694	27,341	36,205
Athlete level (aggregated across events)	153	6214	9995	19,838	35,240	48,962	142,960
Expected‐performance quartiles (athlete‐event level)							
Q1 (lowest expected percentile)	74	5146	8994	15,862	23,871	28,990	35,611
Q2	73	5612	10,402	17,893	26,784	31,944	36,205
Q3	73	4732	7931	13,665	20,118	24,970	34,018
Q4 (highest expected percentile)	74	0	0	7941	14,283	18,354	23,054

∗ This table reports expected financial losses under an elevated commercial value specification (*B* = $30,000), with Operation Gold and stipend components unchanged from the primary analysis and commercial losses scaled linearly. Quartiles are defined by expected percentile finish among health‐affected athlete‐event entries. The overall shape of the distribution remains highly right‐skewed, with substantially larger upper‐tail losses under higher commercial value assumptions.

### Cost‐Effectiveness of Enhanced Illness Prevention Program

#### Baseline Health‐Event Probabilities and Financial Losses

Among Team USA athlete‐event entries (N = 808), 186 were coded as injuries and 108 as illnesses, corresponding to empirically calibrated Markov state probabilities of 0.230 for injury and 0.134 for illness. Under the primary commercial value specification (*B* = $10,000), the total injury/illness‐attributable financial loss across all health‐affected athlete‐event entries was $1,014,210. Losses attributable to injuries totaled $462,409, and losses attributable to illnesses totaled $551,802, consistent with a higher average loss per illness event. Mean athlete‐event loss was $2486 per injury event and $5109 per illness event. These results were consistent with the primary analysis.

#### Base‐Case Cost‐Effectiveness (Team USA Level)

Under the base‐case assumption that the illness prevention program reduces illness risk by 40% and costs $200 per athlete, the program was cost‐saving. Total expected losses averted were $220,721, total program cost was $117,000, and net savings were $103,721 (Table 7). The benefit‐cost ratio was 1.89, indicating that each $1 invested returned $1.89 in avoided losses. The implied incremental cost‐effectiveness ratio was $0.53 per $1 loss averted (i.e., approximately $0.53 spent to avert $1 of expected loss), reflecting financial dominance (net savings > 0).

**TABLE 7:** Base‐Case Cost‐Effectiveness Results and Probabilistic Sensitivity Analysis Summary

Parameter	Value
Prevention program	Enhanced respiratory illness prevention program
Perspective	Team USA (organizational financial loss perspective)
Time horizon	Paris 2024 Olympic Games
Commercial value base (*B*)	$10,000
Number of team USA athletes	585
Number of team USA athlete‐event entries	808
Mean athlete‐event exposures per athlete	1.38
Baseline total financial loss (USD)	$1,014,210
Baseline illness‐attributable loss (USD)	$551,802
Baseline injury‐attributable loss (USD)	$462,409
Assumed illness relative risk reduction (RRR)	40%
Program cost per athlete (USD)	$200
Total program cost (USD)	$117,000
Total financial loss averted (USD)	$220,721
Net savings (USD)	$103,721
Benefit‐cost ratio	1.89
Cost per $1 loss averted (ICER)	$0.53
Loss averted per athlete (USD)	$377
Net savings per athlete (USD)	$177

Probabilistic Sensitivity Analysis (20,000 Simulations)	
PSA Metric	Value
RRR distribution	Beta (9.2, 13.8) (mean 0.40, SD 0.10)
Cost per athlete distribution	Gamma (*k* = 16.0, *θ* = 12.5) (mean $200, SD $50)
Probability intervention is cost‐saving	95.2%
Median net savings (USD)	$102,905
2.5th percentile net savings (USD)	−$17,018
97.5th percentile net savings (USD)	$227,989

ICER, incremental cost‐effectiveness ratio; PSA, probabilistic sensitivity analysis.

#### Base‐Case Cost‐Effectiveness (Athlete Level)

Across Team USA athletes (N = 585), the mean number of athlete‐event exposures per athlete was 1.38. Under base‐case assumptions, the program averted $377 in expected losses per athlete and produced $177 in net savings per athlete after accounting for the $200 program cost. These results indicate that, on average, the program is cost‐saving at both aggregate and per‐athlete levels, although heterogeneity was substantial, as described below.

#### Heterogeneity by Expected‐Performance Tier

Expected net savings varied across expected‐performance quartiles because of differences in mean illness‐associated financial exposure (Table 8). Under the base‐case program parameters, estimated per‐athlete net savings were $162 in Q1, $324 in Q2, $284 in Q3, and −$167 in Q4. This pattern reflects markedly lower mean illness‐attributable losses in Q4 within the fitted performance‐penalty framework. These results support a targeted implementation approach when feasible: deploying the intervention preferentially among athletes with greater expected financial exposure yields substantially higher expected returns.

**TABLE 8:** Expected Per‐Athlete Net Savings by Expected‐Performance Quartile∗

Expected‐Performance Quartile	Mean Illness‐Attributable Loss Per Event (USD)	Expected Loss Averted Per Athlete (USD)	Net Savings Per Athlete (USD)
Q1 (lowest expected percentile)	$3124	$362	$162
Q2	$4118	$524	$324
Q3	$3896	$484	$284
Q4 (highest expected percentile)	$1286	$33	−$167

∗ Expected loss averted per athlete is calculated as the product of mean athlete‐event exposures per athlete (1.38), illness probability, assumed illness relative risk reduction (40%), and quartile‐specific mean illness‐attributable loss. Net savings subtract the fixed program cost of $200 per athlete.

#### Probabilistic Sensitivity Analysis

In probabilistic sensitivity analysis (20,000 iterations), the probability that the program was cost‐saving (net savings > 0) was 95.2% (Figure 7). The median net savings were $102,905, with a 95% uncertainty interval from −$17,018 to $227,989. The cost‐effectiveness acceptability curve indicated that even at a willingness‐to‐pay of $1 per $1 loss averted (financial break‐even), the intervention was cost‐effective in most simulations (Figure 8).

**FIGURE 7:**
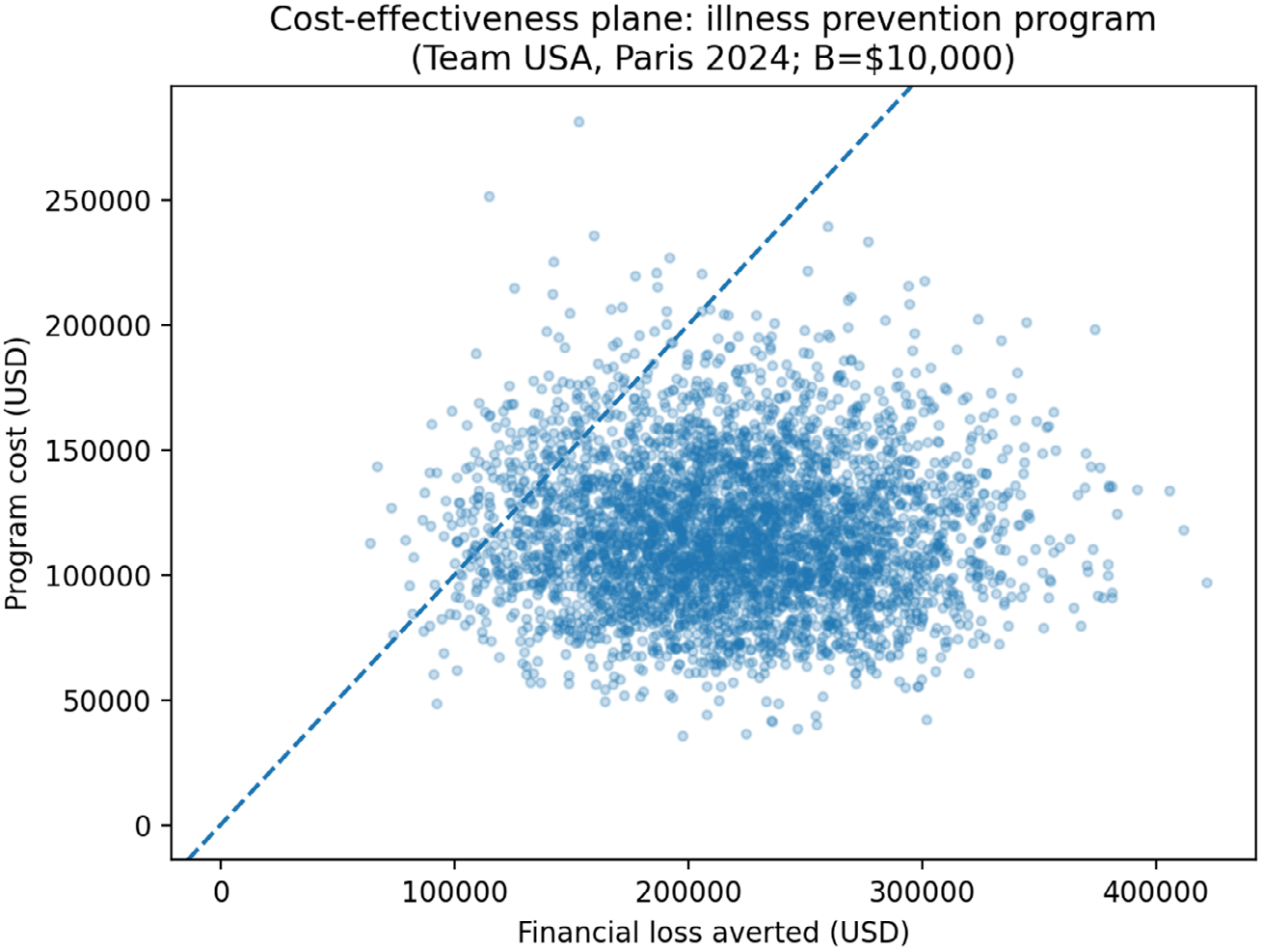
Cost‐effectiveness plane for the illness prevention program cost‐effectiveness plane from the probabilistic sensitivity analysis (20,000 simulations) showing total financial loss averted (*x*‐axis) versus total program cost (*y*‐axis) for the enhanced respiratory illness prevention program among Team USA athletes at the Paris 2024 Olympic Games (commercial base value *B* = $10,000). Each point represents 1 Monte Carlo draw from the joint uncertainty distributions for illness risk reduction and program cost. The dashed diagonal line indicates the threshold where program cost equals financial losses averted (net savings = 0). Points falling below the diagonal represent simulations in which the intervention is cost‐saving.

**FIGURE 8:**
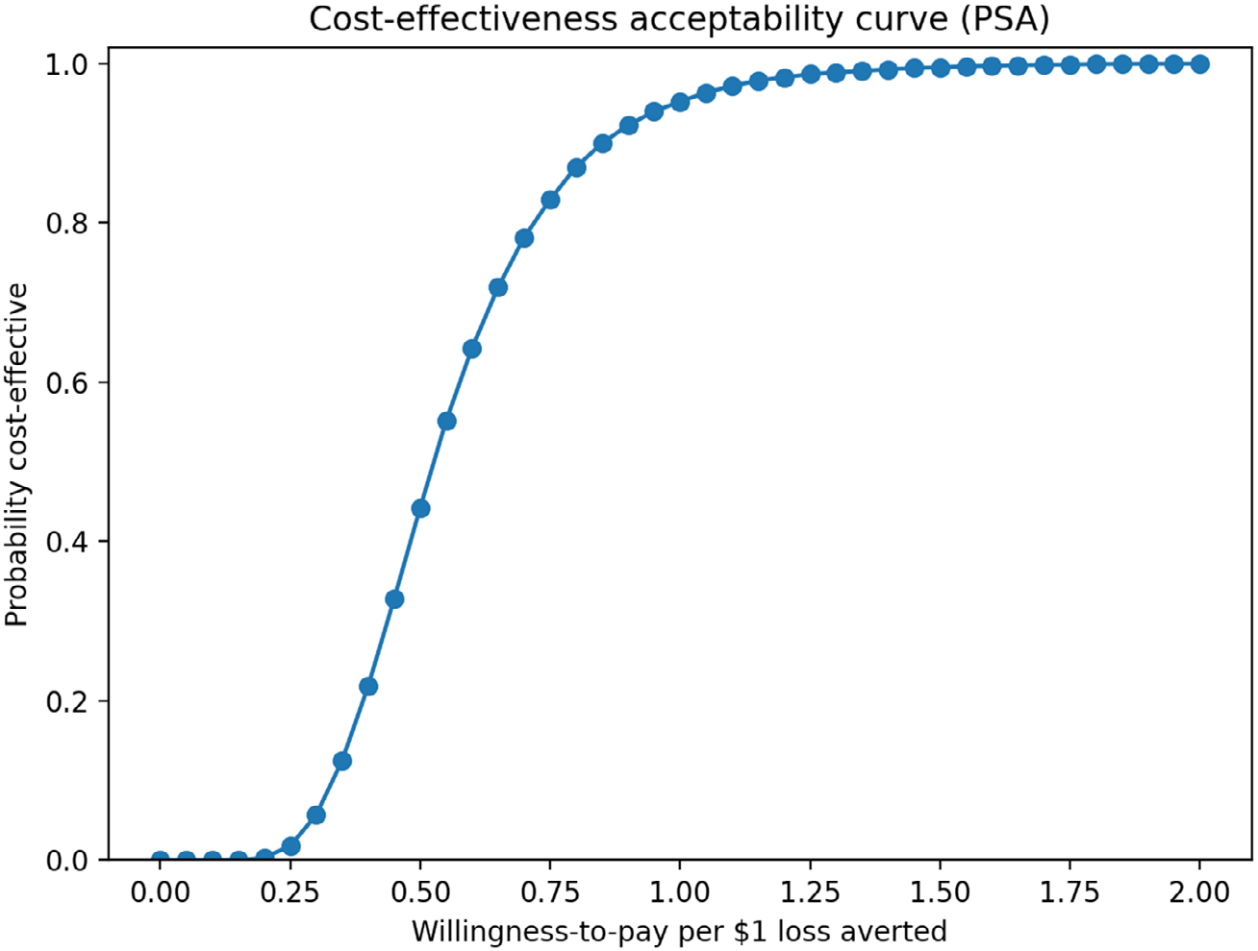
Cost‐effectiveness acceptability curve for the illness prevention program cost‐effectiveness acceptability curve derived from the probabilistic sensitivity analysis, showing the probability that the illness prevention program is cost‐effective across a range of willingness‐to‐pay thresholds per $1 of financial loss averted. At a willingness to pay of $1 per $1 loss averted (financial break‐even), the intervention is cost‐effective in most simulations, indicating a high likelihood of financial dominance under plausible assumptions. (PSA, probabilistic sensitivity analysis.)

The distribution of net savings across the probabilistic simulations is shown in Figure 9. Net savings are centered well above 0, with a right‐skewed distribution reflecting scenarios in which relatively small program costs coincide with large reductions in illness‐related financial losses.

**FIGURE 9:**
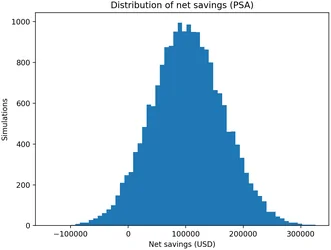
Distribution of net savings from the illness prevention program histogram of net savings (financial losses averted minus program cost) across 20,000 probabilistic sensitivity analysis simulations for the illness prevention program (commercial base value *B* = $10,000). The distribution is centered well above 0, with a long right tail reflecting scenarios in which modest program costs coincide with large reductions in illness‐related financial losses. The small proportion of negative values reflects uncertainty in intervention effectiveness and cost but does not materially alter the overall cost‐saving conclusion. (PSA, probabilistic sensitivity analysis.)

Overall, a feasible illness prevention program was cost‐saving in the base case and remained highly likely to be cost‐saving under probabilistic uncertainty. These findings reflect 3 key features of the observed financial loss structure: (1) illness events were less frequent than injuries but carried substantially higher mean losses per event, (2) prevention benefits scale with the magnitude of illness‐attributable financial exposure, and (3) the right‐skewed distribution of losses creates substantial upside for prevention programs that reduce illness incidence even modestly.

## DISCUSSION

The most important finding of this study was that, among 808 Team USA athlete‐event entries, injury and illness were associated with approximately $1.01 million in estimated financial losses that were concentrated among a small subset of athletes, with illnesses accounting for a greater share of losses than injuries and the modeled illness‐prevention program being cost‐saving in 95.2% of simulations. More specifically, 4 primary findings emerged. First, injury and illness were associated with substantial expected financial losses when performance decrements were evaluated relative to precompetition expectations. Second, financial losses were highly concentrated, with a small proportion of athletes accounting for a disproportionate share of the total financial impact, consistent with the threshold‐driven nature of elite sport incentives. Third, illnesses accounted for a larger share of total financial losses than injuries, despite occurring less frequently, aligning with prior evidence that respiratory and other illnesses can meaningfully disrupt readiness during major Games settings.^6^, ^12^ Fourth, a feasible illness prevention program was likely to be cost‐saving at both the aggregate and athlete levels under a wide range of assumptions, consistent with consensus guidance emphasizing surveillance‐informed prevention and targeted risk reduction strategies in elite sport.^1^, ^12^

The first major finding was that injury and illness translated into meaningful expected financial losses through multiple pathways, including performance incentives, stipend eligibility, and commercial value. This extends prior Olympic and Team USA surveillance work that has traditionally emphasized incidence, sport variation, and clinical characteristics of injuries and illnesses during major multisport competitions.^2^, ^3^, ^4^, ^5^, ^6^, ^7^, ^8^, ^9^, ^11^, ^12^ By linking health events to expectation‐based performance counterfactuals, the present findings complement recent Paris‐specific evidence showing systematic underperformance among athletes experiencing injury or illness during the Games period.^10^

A second key finding was the extreme concentration of financial losses. Although median athlete‐level losses were relatively modest, the upper tail of the distribution was substantial, with a small subset of athletes accounting for most total losses. This pattern is consistent with the nonlinear structure of competition and reward, in which minor rank shifts near medal or qualification thresholds can produce outsized changes in rewards and downstream opportunities.^15^, ^17^ From the sport organization health care provider perspective, consistent vigilance to adherence of illness prevention efforts, especially among top medal contenders, could have significant financial and commercial benefit to the individual and collective group of athletes. The concentration of losses also aligns with broader evidence that compensation structures in sport can generate substantial heterogeneity in perceived economic impact across athletes, particularly when bonus‐ and subsidy‐like mechanisms are salient.^17^

Third, illnesses accounted for a larger proportion of total financial losses than injuries. This occurred despite injuries being more common and appears consistent with prior Team USA and multisport evidence that respiratory and other acute illnesses can cluster around travel and early‐Games exposure windows and may disproportionately affect readiness in ways that are not fully captured by time‐loss classification alone.^6^, ^12^ In addition, illness‐attributable losses may be amplified when illness episodes overlap with athletes who have higher ex ante podium probabilities, thereby increasing exposure to threshold‐based incentive displacement.

Finally, a cost‐effectiveness analysis showed that an illness prevention program was likely to reduce financial losses in a cost‐saving manner. This finding is directionally consistent with International Olympic Committee consensus recommendations that emphasize the central role of surveillance, high‐quality data capture, and structured prevention initiatives to protect athlete health and mitigate downstream consequences.^1^ Given evidence that illness epidemiology at the Games is context‐specific but recurrently features respiratory illness as a leading category, targeted prevention approaches that are followed by athletes and staff remain a plausible lever for reducing meaningful performance and financial disruption during major competitions. This finding may also provide additional perspectives for discussions between athletes and staff that despite perceived inconvenience of illness prevention programs while at the Olympic games, adherence to these programs supports higher performance and financial outcomes.

Several methodological strengths support confidence in these findings. The use of precompetition performance expectations allowed health‐related underperformance to be quantified relative to a realistic counterfactual rather than relying solely on observed placements. In addition, the reliance on structured surveillance methods aligns with best‐practice guidance for injury/illness data quality and reporting consistency.

### Limitations

This study has several limitations. Commercial‐value estimates relied on simplifying assumptions and did not capture financial consequences extending beyond the Olympic Games. The analyses did not account for the severity, timing, duration, or specific diagnosis of each injury or illness, which may influence its effect on performance. Expected outcomes and health‐related performance decrements were model‐based and may be affected by residual confounding or misspecification of the counterfactual performance distributions. Finally, the findings were derived from Team USA athletes at a single Olympic Games and may not generalize to athletes from other countries, competitions, or financial support systems. Sex‐disaggregated outcome analyses were not performed because the number of health‐affected athlete‐event observations within sex‐specific sport and event strata was insufficient to support stable and interpretable estimates.

## CONCLUSIONS

Injury and illness at the Olympic Games are associated with substantial and highly concentrated financial losses, with illnesses accounting for a disproportionate share of the total impact on Team USA athletes.

## SUPPORTING INFORMATION

Additional supporting information can be found online in the Supporting Information section.

## DISCLOSURES

The authors (J.F.O., T.A., A.N.T., A.D., J.T.F., H.K.V., E.G.P.) declare that they have no known competing financial interests or personal relationships that could have appeared to influence the work reported in this paper.

## Supporting information

Supplementary Material
