# Supplementary material for: Health‐Related Performance Decrements Resulted in Substantial Financial Loss for Team USA Athletes at the Paris 2024 Olympic Games
Source: Arthrosc Sports Med Rehabil. 2026 Oct 27:e70132. Online ahead of print. doi: 10.1002/ars2.70132 (PMC13712391; doi:10.1002/ars2.70132)
Supplement: Supplementary file 1 [file ARS2-9999-e70132-s001.pdf]

Appendix I

Pairwise Calibration of Win Probability to Latent Strength

The value p_i denoted competitor i's implied win probability. A latent strength score was defined as $s_i = \text{logit}(p_i)$. To convert these strengths into a full ranking distribution, a single slope parameter α was estimated from all events by maximum likelihood under a pairwise-ordering model:

$$Pr(i\ beats\ j) = logit^{-1}(\alpha(s_i - s_j)),$$

using all within-event ordered pairs consistent with observed rankings (better observed rank implies "beats"). This calibration step learns how differences in logit (win probability) translate into differences in head-to-head superiority across the Olympic field.

Markov Ranking Model and Monte Carlo Inference

Event outcomes were modeled as a sequential selection process equivalent to a Plackett–Luce model implemented as a Markov process. At step t, the next finishing position was drawn from the remaining competitors with probability proportional to $\exp(\alpha s_i)$. The state of the process was the set of remaining competitors, and transitions correspond to selecting the next finisher. For each event, 5,000 Monte Carlo realizations of this Markov ranking process were generated to estimate each Team USA competitor's *ex ante* distribution of final rank. Because competitive deduplication can make the simulated field size differ from the event's observed rank maximum, simulated positions were mapped back to the event rank scale using a monotone linear mapping so that medal and top-K probabilities correspond to the placement scale. Expected percentile finish was computed as $100 \cdot (N - r)/(N - 1)$, where r was rank and N was the event rank maximum.

Injury/Illness Performance Penalty Estimation

Percentile performance was modeled as a function of market expectation and injury/illness using:

$$\text{ObservedPercentile} = \beta 0 + \beta 1 \cdot \text{ExpectedPercentile} + \beta 2 \cdot \text{Injury} + \beta 3 \cdot (\text{ExpectedPercentile} \cdot \text{Injury}) + \varepsilon.$$

Inference used heteroscedasticity-consistent (HC3) standard errors. A robust Huber M-estimator regression was fit as a sensitivity analysis.

Counterfactual Injury-Adjusted Performance Distributions

For each injured athlete–event, the fitted model was used to compute the expected percentile under injury/illness and under a counterfactual no-injury condition (injury indicator set to 0, holding expected percentile fixed). The implied percentile decrement was converted to a rank shift on the event's rank scale via $\Delta r = -\Delta \text{percentile} \cdot (N - 1)/100$, truncated at 0 so that injury/illness could not improve rank in the counterfactual adjustment step. This rank-shift penalty was then applied directly to each simulated draw of the athlete's rank distribution ($\text{rank}_{\text{draw}} + \Delta r$), producing injury-adjusted medal and top-K probabilities, while preserving the event-specific uncertainty from the Markov ranking process.

Appendix II

Decomposition of Financial Losses into Injury-Attributable vs. Illness-Attributable Components

To quantify the proportion of financial losses attributable to injuries versus illnesses, injury and illness were modeled as separate exposures rather than a single combined indicator. Specifically, observed percentile finish was regressed on expected (*ex ante*) percentile finish with separate main effects and interaction terms for injury and illness:

$$\text{ObservedPercentile} = \beta 0 + \beta 1 \cdot \text{ExpectedPercentile} + \beta 2 \cdot \text{Injury} + \beta 3 \cdot \text{Illness} + \beta 4 \cdot (\text{ExpectedPercentile x Injury}) + \beta 5 \cdot (\text{ExpectedPercentile x Illness}).$$

Heteroscedasticity-consistent (HC3) standard errors were used for inference. The fitted marginal effect of injury at expected percentile E was defined as $\Delta_{Injury}(E) = \beta 2 + \beta 4 \cdot E$, and the fitted marginal effect of illness was defined as $\Delta_{Illness}(E) = \beta 3 + \beta 5 \cdot E$.

For each athlete–event entry, the event-specific Markov ranking simulations yielded a baseline (no health-event) distribution of placements. For entries labeled as injuries, an injury-specific rank penalty was applied to each simulated placement draw; for entries labeled as illnesses, an illness-specific rank penalty was applied. Rank penalties were derived by mapping the estimated percentile decrement to the event's rank scale:

$$RankShift = max(0, -\Delta(E) \cdot (N-1)/100),$$

where N is event field size. This truncation ensured health events could worsen but not improve simulated performance. The injury- or illness-adjusted simulated placement distributions were then used to recompute expected Operation Gold, stipend, and commercial value outcomes. Injury-attributable losses were computed as the sum over injury-labeled athlete–event entries of the difference between baseline (no-health-event) expected value and injury-adjusted expected value. Illness-attributable losses were computed analogously for illness-labeled entries. Percent contributions were calculated as:

$$\%Injury = Loss_{Injury} / (Loss_{Injury} + Loss_{Illness}) \times 100,$$
$$\%Illness = Loss_{Illness} / (Loss_{Injury} + Loss_{Illness}) \times 100.$$

This decomposition was repeated across commercial base value parameters B = \$3,000, \$10,000, and \$30,000 to verify robustness of attribution.

Appendix III

Markov Structure and Transition Probabilities

The Markov model included the following states: No health event, Injury, and Illness. State membership was assigned per athlete–event exposure using the observed frequencies at the 2024 Paris Olympic Games. Transition probabilities were therefore calibrated empirically as:

$$p(Injury) = proportion\ of\ Team\ USA\ athlete - event\ entries\ coded\ as\ injuries,$$
$$p(Illness) = proportion\ coded\ as\ illnesses,$$
$$p(No\ event) = 1 - p(Injury) - p(Illness)$$

Because the model is defined at the athlete–event unit, the cohort expected value represents aggregate impact across the observed Team USA athlete–event portfolio.

Costs (Financial Losses)

Each adverse health state (Injury or Illness) was assigned a state-dependent expected loss, estimated from the injury/illness-adjusted simulated placement distributions and financial valuation pipeline. Specifically, the expected cost for injuries was the mean athlete–event loss among injury-coded athlete–event entries; the expected cost for illnesses was the analogous mean among illness-coded entries. Expected losses included the combined expected reduction in Operation Gold incentives, stipend eligibility, and commercial value (commercial base value parameter B = $10,000 in the primary analysis). The baseline expected financial loss per athlete–event was computed as:

$$E[Loss] = p(Injury) \cdot \bar{L}_{Injury} + p(Illness) \cdot \bar{L}_{Illness}.$$

Total Team USA expected loss was computed by multiplying the expected loss per athlete–event by the total number of Team USA athlete–event entries at the 2024 Paris Olympic Games.

Prevention Program and Effect Assumptions

An illustrative prevention program was selected to be operationally feasible in an Olympic village setting and supported by established infectious disease prevention principles: an Enhanced Respiratory Illness Prevention Bundle (e.g., vaccination optimization, rapid testing and isolation protocols, portable HEPA filtration in high-density indoor spaces, masking during outbreaks/high-risk travel, and hygiene reinforcement). The intervention was modeled as reducing the probability of illness at each athlete–event exposure by a relative risk reduction (RRR) while leaving injury risk unchanged:

$$p'(Illness) = p(Illness) \cdot (1 - RRR).$$

The intervention cost was modeled as a fixed per-athlete program cost, applied to all Team USA athletes.

Outcomes and Cost-Effectiveness Metrics

Primary outcomes included financial loss averted (USD) at the Team USA level, net savings = loss averted − program cost (USD), benefit–cost ratio = loss averted / program cost, and per-athlete expected savings and per-athlete net savings based on the observed athlete–event exposures per athlete.

Because outcomes and costs are both in USD, cost-effectiveness was interpreted under a financial dominance framework: the program was considered cost-effective if expected savings exceeded program cost (net savings > 0). An ICER was also computed as cost per $1 loss averted.

Heterogeneity and Tier-Based Analysis

To examine effect heterogeneity, per-athlete expected net savings were estimated within expected-performance quartiles. Quartile-specific mean illness-attributable losses were computed from the athlete–event loss dataset, and illness-prevention benefits were calculated under the assumption of equal illness-risk reduction applied across tiers.

Probabilistic Sensitivity Analysis

A probabilistic sensitivity analysis (PSA) was performed to quantify uncertainty in intervention cost-effectiveness. RRR was modeled using a Beta distribution with mean 0.40 and standard deviation 0.10, and program cost per athlete was modeled using a Gamma distribution with mean $200 and SD $50. For each of 20,000 PSA iterations, total savings, total cost, net savings, and the probability of cost-saving were computed.
